# Reexamining a Host-Associated Genomic Diversity of Bean Golden Mosaic Virus (BGMV) Isolates from *Phaseolus* Species and Other Fabaceae Hosts

**DOI:** 10.3390/pathogens14070697

**Published:** 2025-07-15

**Authors:** Luciane de Nazaré Almeida dos Reis, Josiane Goulart Batista, Maria Luiza Fernandes de Oliveira, Maria Esther de Noronha Fonseca, Josias Corrêa de Faria, Francisco José Lima Aragão, Leonardo Silva Boiteux, Rita de Cássia Pereira-Carvalho

**Affiliations:** 1 Departamento de Fitopatologia, Universidade de Brasília (UnB), Brasília 70910-900, DF, Brazil; lucianealmeidareis@outlook.com (L.d.N.A.d.R.); 05josiane@gmail.com (J.G.B.); mlferoliver20@gmail.com (M.L.F.d.O.); 2 Embrapa Hortaliças, National Center for Vegetable Crops Research (CNPH), Brasília 70351-970, DF, Brazil; maria.boiteux@embrapa.br; 3 Embrapa Arroz e Feijão, Goiânia 75375-000, GO, Brazil; josias.faria@embrapa.br; 4 Embrapa Cenargen, Laboratório de Expressão de Genes, Brasília 70770-917, DF, Brazil; francisco.aragao@embrapa.br

**Keywords:** *Begomovirus*, common bean, lima bean, sequence demarcation tool

## Abstract

Beans *(Phaseolus vulgaris* and *P. lunatus*) are the major hosts of bean golden mosaic begomovirus (BGMV). Robust taxonomic criteria were established for *Begomovirus* species demarcation. However, DNA–A identities among BGMV isolates display a continuous variation (89–100%), which conflicts with the current concept of a single viral species. The diversity of 146 Brazilian isolates designated in the GenBank as BGMV was assessed by comparing their complete DNA–A sequences. The isolates were clustered into four groups, being discriminated mainly by their original Fabaceae hosts. Additional Sequence Demarcation Tool analyses indicated that BGMV-related viruses comprise two clear-cut groups: isolates reported infecting mainly *P. vulgaris* (identities of 96–97% to the reference NC_004042 isolate) and a group associated with *P. lunatus* (identities of 89–91%). Moreover, we recognized a distinct set of genomic features in the iterons and Rep-associated protein motifs across these two diversity groups. The host prevalence and genomic differences suggest that most *P. lunatus* isolates are currently misclassified as BGMV strains, being more likely samples of a closely related (but distinct) *Begomovirus* species. Hence, the implications of this BGMV diversity should be taken into consideration by classical and biotech breeding programs aiming for large-spectrum viral resistance in *Phaseolus* species.

## 1. Introduction

Viral species of the family *Geminiviridae* are responsible for significant yield and quality losses in several economically important vegetable and field crops, including *Phaseolus* species and other Fabaceae hosts [1]. *Begomovirus* is the largest genus within this family with over 400 species described thus far [2]. Begomoviruses are characterized by circular, single–stranded (ss) DNA genomes, encapsulated in twinned icosahedral particles (18–20 × 30–32 nm). These viruses can have either only one (=monopartite) or two (=bipartite) DNA components [3]. Begomoviruses are efficiently transmitted by members of the *Bemisia tabaci* (Hemiptera: Aleyrodidae) cryptic species complex, being able to infect a wide range of dicot hosts [2,3]. Due to the increasing number of viruses that have been characterized within the genus *Begomovirus*, robust sets of taxonomic rules have been established in recent years for novel species demarcation [2]. In the first set of taxonomical criteria, a new species was defined when the nucleotide identity levels of the complete DNA–A component was less than 89% in comparison with all the available viral sequences [4]. Subsequently, a novel standardized set of criteria was elaborated that requires comparative analyses employing multiple MUSCLE alignments in combination with Sequence Demarcation Tool—SDT [3] analysis. In the current classification system, a novel species can only be defined when the nucleotide identity of the entire DNA–A component displays less than 91% in comparison with the complete DNA–A genome of any other previously described *Begomovirus* species. If a given DNA–A sequence shares above 94% identity with the complete DNA–A genome, the virus is then classified as a viral strain [3].

Bean golden mosaic virus—BGMV (*Begomovirus costai*) and bean golden yellow mosaic virus—BGYMV (*Begomovirus birdi*) are the most important bean-infecting begomoviruses in the Americas and in the Caribbean region, being the causal agents of the “bean golden mosaic disease” [5,6,7]. Up to recently, BGMV was the only begomovirus infecting common beans (*Phaseolus vulgaris* L.) and lima beans (*P. lunatus* L.) in Brazil [5,7,8]. However, the weed-associated Macroptilium yellow spot virus (MacYSV; *Begomovirus macroptilimaculae*) has also been reported as an emergent pathogen of beans in northeast region of Brazil [9,10]. Nevertheless, BGMV is still very important in that geographic region [10]. In many traditional growing regions, the bean cultivation has become almost unfeasible due to the high levels of BGMV incidence [5]. BGMV isolates infecting other legume hosts such as *Glycine max* (L.) Merr. and *Macroptilium lathyroides* (L.) Urb. have also been described after the invasion of *B. tabaci* Middle East-Asia Minor 1 (MEAM 1 = biotype B) in the early 1990s [9,10,11]. More recently, BGMV has also been described infecting the legume weed *M. erythroloma* (Mart. ex Benth.) in Brazil [12].

Studies dealing with the BGMV diversity in *Phaseolus* species as well as in other legume hosts have been conducted under Brazilian conditions [8,10,13]. Overall, the results indicated relatively low genetic variability among BGMV populations [10]. However, a peculiar host-associated genetic diversity was observed across isolates from *P. vulgaris* and *P. lunatus*. We then carried out a preliminary examination of the DNA–A identities across isolates designated as either BGMV or BGMV strains available in public databases, and we observed a continuous variation (89–100%), which appears in conflict with the currently established taxonomic criteria for a single viral species in the genus *Begomovirus* [3]. In fact, a previous observation suggested the presence of a putative novel viral species (distinct from BGMV) in association with lima beans [8]. The name lima bean golden mosaic virus (LBGMV) was suggested for this divergent isolate [8]. However, this nomenclature was not adopted probably because this initial viral description was performed only with a partial genomic sequence of 1185 nucleotides (= GenBank U92531) encompassing a segment coding for the Rep protein (Rep), common region (CR), and the coat protein (CP). In addition, the standard taxonomic rules for novel *Begomovirus* species demarcation were not well established at that time [3,4].

Due to the economic and biological significance of the bean–BGMV pathosystem, we decided to carry out a more extensive analysis in order to catalog the genetic variability of all available isolates from Brazil classified as either BGMV or BGMV strains from *Phaseolus* species as well as other Fabaceae hosts.

## 2. Materials and Methods

**Figure 1:**
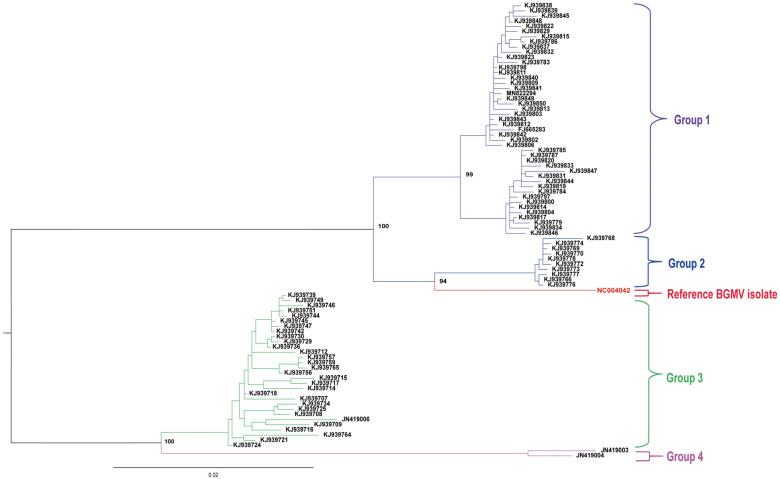
Phylogenetic tree of a set of complete genomes of DNA–A components showing the phylogenetic identities/distances of a subset of 88 out of 146 bean golden mosaic virus (BGMV) isolates available at the GenBank. Midpoint–rooted ML with 1000 bootstrap replications. Group #1 was composed by BGMV isolates reported infecting *Phaseolus vulgaris*, soybean (*Glycine max*) and *Macroptilium erythroloma* (with branches in purple), GenBank accessions (Supplementary Table S1); Group #2 was composed of BGMV isolates obtained from *M. lathyroides* (with branches in blue); Group #3 was composed of BGMV isolates obtained from *P. lunatus* (with branches in green); Group #4 was composed by two highly divergent BGMV isolates reported infecting the weed *M. lathyroides* (with branches in pink). The complete set of information for all 146 isolates is provided in Supplementary Table S1.

**Figure 2:**
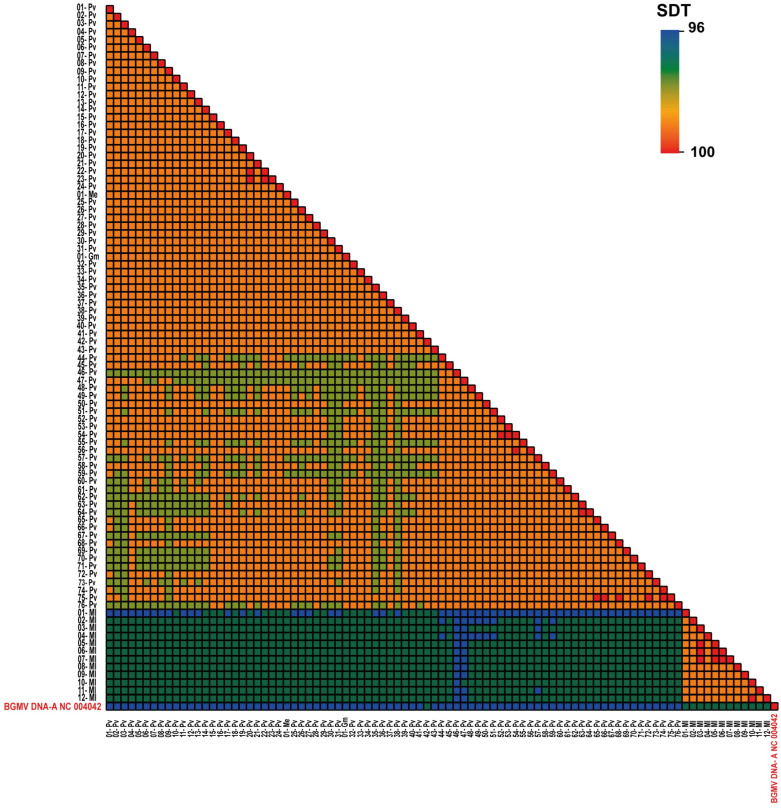
Pairwise identity analysis with the Sequence Demarcation Tool (SDT) was carried out using the sequence information of the DNA–A component of a subset of 88 out of 146 isolates obtained from *Phaseolus vulgaris*, *Macroptilium lathyroides*, *M. erythroloma*, and *Glycine max* indicating their identities in relation to the sequence of the reference (NC_004042) bean golden mosaic virus (BGMV) isolate (highlighted in red font color). BGMV isolates from *Phaseolus vulgaris* are identified by a numerical order and they correspond to the following GenBank accessions: *Phaseolus vulgaris* isolates (Pv)*:* 01 (KJ939839), 02 (KJ939838), 03 (KJ939810), 04 (KJ939848), 05 (KJ939829), 06 (KJ939836), 07 (KJ939786), 08 (KJ939815), 09 (KJ939845), 10 (KJ939837), 11 (KJ939822), 12 (KJ939824), 13 (KJ939832), 14 (KJ939823), 15 (KJ939811), 16 (KJ939798), 17 (KJ939841), 18 (KJ939809), 19 (KJ939816), 20 (KJ939801), 21 (KJ939805), 22 (KJ939795), 23 (KJ939813), 24 (KJ939849), 25 (KJ939852), 26 (KJ939818), 27 (KJ939781), 28 (KJ939840), 29 (KJ939783), 30 (KJ939782), 31 (KJ939803), 32 (KJ939842), 33 (KJ939853), 34 (KJ939793), 35 (KJ939812), 36 (MG334552), 37 (KJ939843), 38 (KJ939851), 39 (KJ939792), 40 (KJ939802), 41 (KJ939850), 42 (KJ939799), 43 (KJ939806), 44 (KJ939844), 45 (KJ939826), 46 (KJ939847), 47 (KJ939835), 48 (KJ939830), 49 (KJ939821), 50 (KJ939831), 51 (KJ939819), 52 (KJ939825), 53 (KJ939827), 54 (KJ939788), 55 (KJ939787), 56 (KJ939785), 57 (KJ939820), 58 (KJ939833), 59 (KJ939828), 60 (KJ939780), 61 (KJ939784), 62 (KJ939790), 63 (KJ939779), 64 (KJ939817), 65 (KJ939800), 66 (KJ939789), 67 (KJ939794), 68 (KJ939807), 69 (KJ939808), 70 (KJ939791), 71 (KJ939796), 72 (KJ939797), 73 (KJ939814), 74 (KJ939804), 75 (KJ939834) and 76 (KJ939846); *Macroptilium erythroloma* isolate (Me) (MN822294); *Glycine max* isolate (Gm); (FJ665283); *Macroptilium lathyroides* isolates (Ml): 01 (KJ939725), 02 (KJ939714), 03 (KJ939707), 04 (KJ939756), 05 (KJ939708), 06 (KJ939732), 07 (KJ939764), 08 (KJ939733), 09 (KJ939709), 10 (KJ939717), 11 (KJ939715), 12 (KJ939734)].

A collection of 146 complete DNA–A genomic sequences of BGMV isolates/strains occurring across major production areas of Brazil was retrieved from the GenBank database (Supplementary Tables S1 and S2). We performed a phylogenetic analysis with all 146 isolates (Supplementary Figure S1). In order to improve the visual clarity of the data, we also carried out a simplified analysis with a subset of 88 isolates that displayed more than 98% of identity among them (Figure 1 and Figure 2). The original hosts of these BGMV isolates were the following: 77 from *P. vulgaris*, 55 from *P. lunatus* as well as one isolate from *G. max*, 12 from *M. lathyroides*, one from *M. erythroloma*. In addition, complete DNA–B genomes were also retrieved from the NCBI database, including two from *P. vulgaris*, one from *P. lunatus*, two from *M. lathyroides*, and one of *M. erythroloma*. Phylogenetic analyzes were carried out employing genomic information of these BGMV isolates with complete sequence of the DNA–A component. The phylogenetic tree was generated from the alignment of the complete DNA–A component of each isolate, using the MUSCLE program implemented by the Geneious^®^ 11.0 program (PhyML method, model F81 with 1000 bootstrap replications). Multiple MUSCLE alignments were performed in SDT v1.2 [14] and the figures were elaborated with Adobe Illustrator CC 2021 and EvolView v3 [15]. Comparative genomic analyses were conducted with the Geneious^®^ 11.0 program [16].

We analyzed the nucleotide sequences of the common region (CR) of the cognate DNA–A and DNA–B components as well as the replication-associated protein (Rep) motifs [17]. In addition, we carried out BLASTn analysis aiming to compare a 412 bp sequence of the Rep gene across BGMV-related isolates. This conserved viral genomic segment was used for the RNAi intron-hairpin construct that resulted in a transgenic BGMV-resistant bean cultivar [18]. Comparative analyses were also conducted using genomic information from two structural elements conserved across several genera of the *Geminiviridae* family [19,20]: the helix-4 motif and the quasi–palindromic DNA–A segment [ACTT–(N7)–AAGT]. The presence as well as genomic features of the ORF AC5 were also investigated across the BGMV-related isolates.

## 3. Results

Phylogenetic analysis of a set of 100 and 146 isolates with complete DNA–A information indicated a clear-cut discrimination into four clusters. These groups of isolates were mainly organized in accordance with their original legume hosts (Figure 1 and Figure S1). Group #1 was composed of BGMV isolates reported infecting mainly *P. vulgaris*, but also *G. max*, *P. lunatus*, and *M. erythroloma*. Group #2 comprised BGMV isolates obtained from *M. lathyroides*, whereas Group #3 encompassed BGMV isolates mainly obtained from *P. lunatus*, but also from unclassified *Phaseolus* species and *M. lathyroides*. Finally, Group #4 was composed of only two divergent BGMV isolates reported infecting *M. lathyroides*.

Using SDT and MUSCLE alignments, including the isolates of Group #1 and Group #2 as well as the DNA–A component of the BGMV reference isolate (NC_004042), showed identity levels ranging from 96 to 97% among them (Figure 2). These results indicated that all these isolates belong to the same viral lineage of the reference BGMV isolate. However, the SDT analyses employing the isolates belonging to Groups #3 and #4 displayed identity levels ranging from 89 to 91% in relation to the reference BGMV isolate (NC_004042). In fact, most of the *bona fide* *P. lunatus* isolates and from unclassified *Phaseolus* species displayed identity levels close to 91% when compared to NC_004042 (Figure 2). Exceptions were observed for three isolates (KJ939711, KJ939710, and KJ939720), whose nucleotide identities ranged from 94 to 95% to representative BGMV isolates (Figure S2). Furthermore, some isolates also classified as BGMV (viz. KJ939735, KJ939731, KJ939719, JF694451, JF694449, and JF694452) displayed identities of 90% (Figure S1), indicating that they are more likely representing isolates of a novel species according to the current criteria for the classification in the genus *Begomovirus* [3]. SDT analyses among the isolates of Groups #3 and #4 displayed identity levels among them ranging from 95 to 99%, indicating that they belong to the same strain as the reference isolate.

Comparisons of a subgroup of BGMV isolates obtained from the weed *M. lathyroides* (clustered in Group #2) displayed identities of around 97% with the reference isolate (NC_004042) (Figure S3). However, a distinct subgroup of *M. lathyroides* isolates (previously classified as BGMV) clustered in Groups #3 and #4 and showed identity levels of 89–90% (e.g., JN419004 and JN419003) and 91% (e.g., JN419006) with representative the BGMV isolates. Therefore, as previously observed [9], *M. lathyroides* seems to be an “universal” host of distinct BGMV-related isolates (Figure S3) as well as from other legume-infecting viral species. When compared to *P. lunatus* isolates, a subgroup of *M. lathyroides* isolates from Groups #3 and #4 displayed identity levels ranging from 95 to 99%, indicating they are more likely isolates of the same viral species (Figure S3). Likewise, the BGMV isolates from *G. max* (FJ665283) and *M. erythroloma* (MN822294), displayed identity ranging from 96 to 97% to representative BGMV sequences (including the reference isolate).

Comparative analyses employing the DNA–B sequences available at GenBank of *P. vulgaris*, *P. lunatus*, unclassified *Phaseolus* species, and *M. erythroloma* isolates indicated overall identities ranging from 89 to 100%. The sequences of isolates obtained from *M. lathyroides* displayed the lowest identity levels (79–82%) when compared to all available DNA–B sequences (Figure S4).

In order to verify the hypothesis that a subgroup of isolates named as BGMV may represent a distinct begomovirus, we also carried out analyses encompassing the common region (CR) of the DNA–A and DNA–B components of all isolates with these available genomes. The iterons of the reference isolate as well as across all BGMV isolates with identity levels greater than 91%, displayed the sequence GGTGT (Rep iteron-related domain—Rep IRD = MPPPKRFKIN) [17]. They were found twice in the CRs and they did not display an inverted sequence. The CRs of the DNA–A and DNA–B components of isolates corresponding to putative new species also showed distinct iterons. The iteron found in the sequences of *P. lunatus*, unclassified *Phaseolus* species, and in a subgroup of the *M. lathyroides* isolates (e.g., JN419003 and JN419006) was GGGGT and the inverted sequence ACCCC (Rep IRD = MPPPKRFKIS), differing from the reference BGMV isolate in the last amino acid residue that was replaced by a serine. An exception was observed in the isolate KJ939719 that showed a distinct Rep IRD (= MPPPKRFRIS). In the DNA–A genome of the *M. lathyroides* isolate (JN419004) and in the corresponding DNA–B genome (JN419017), the iteron GGTAC and its inverted GTACC sequence (Rep IRD = MPPPKRFKIS) were found.

We also examined potential differences across isolates classified as BGMV for the structural helix-4 motif, whose amino acid sequence is strongly conserved across geminiviruses [19]. The sequences of the BGMV isolates compared to the reference isolate showed minor amino acid differences. All isolates differed with the reference isolate at the 184th position. The polar amino acid tyrosine (Y) is present in the reference isolate, whereas in other isolates this residue was replaced by the non–polar amino acid phenylalanine (F) (Figure S5). Other BGMV isolates showed additional (but not biologically relevant) residue differences at positions #167 (e.g., isolates of *M. lathyroides*) as well as #175 and #178 (e.g., the isolate KJ939720 from *P. lunatus*). Isolates previously classified as BGMV when compared with reference BGMV isolate also displayed differences in the 184th position (Figure S6). Another difference in relation to the BGMV reference sequence was at position 175th in which the basic amino acid lysine (K) is present. However, most of the other sequences displayed the non-polar amino acid proline (P), except for the sequences of *M. lathyroides* (JN419004 and JN419003), which displayed a polar amino acid glutamine (G) (Figure S6, panel A). A subgroup of isolates showed distinct but not significant differences at positions #171, #197, and #198, including *M. lathyroides* (JN419004 and JN419003) and #181 (KJ939735 and KJ939731 isolates from *P. lunatus*). The highlighted amino acid residues in Figure S6 (panel B) are the ones predicted to interact with the plant retinoblastoma-related protein (pRBR) to modulate the overall host gene expression [19].

We also carried out analyses the quasi-palindromic DNA–A segment [ACTT–(N7)–AAGT] that is a structural element conserved across the coat protein (CP) gene promoter of several members of the *Geminiviridae* family [20]. The sequences of the BGMV isolates as well as of the reference isolate are illustrated in Figure S7. Analyses showed motif diversity among BGMV isolates when compared with the reference isolate (NC_004042), which has the motif ACTT–GTCGCCC–AAGT. Overall, the motif sequences found were GGCGACC, GGTGACC, GGCGACC, GGCAACC, GGTGTCC, and GGCCCCC. These differences were detected mainly in the second to the fifth positions (Figure S4). Seventy-three (73) isolates from *P. vulgaris* displayed the sequence GGCGACC, except for the sequences of *P. vulgaris* isolates KJ939851 and KJ939795 that displayed the sequence GGTGACC with differences in the third position. Three isolates from *P. lunatus* (KJ939710, KJ939720, and KJ939719) shared identities greater than 96% with the reference BGMV, displayed the sequence GGTGTCC, except for the KJ939719 isolate, which displayed the sequence GGCCCCC. Twelve isolates from *M. lathyroides* (with identity levels of 97% to BGMV) displayed the sequence GGCAACC. On the other hand, isolates obtained from *M. erythroloma,* and *G. max* displayed the sequence GGCGACC. The sequences found in isolates classified as BGMV were ACTT–GGCCCCC–AAGT, apart from a subgroup of isolates that displayed the alternative sequences (viz. GACCCTC, GGCCCCG, and GGCCCCTC), with differences in the second, sixth, and seventh positions. Thirty (30) isolates from *P. lunatus* and all isolates from an unclassified *Phaseolus* species (with identity levels ranging from 90% to 91% with the reference BGMV isolate) displayed the GGCCCCC sequence. Exceptions were found, however, in six *P. lunatus* isolates (KJ939731; KJ939764; KJ939707; KJ939721; KJ939722; KJ939723) in which the GACCCTC sequence was annotated. Exceptions were also found in a group of 17 isolates (KJ939746; KJ939752; KJ939738; KJ939749; KJ939744; KJ939751; KJ939748; KJ939739; KJ939743; KJ939741; KJ939737; KJ939750; KJ939747; KJ939740; KJ939742; KJ939745; KJ939753) that displayed the alternative GGCCCCG sequence. Three isolates from *M. lathyroides* classified as BGMV (with identity levels of 89–91%) displayed the alternative sequences GGCCCCTC (JN419006) and GGCCCCC (JN419004 and JN419003).

The ORF AC5 has been identified in a subgroup of begomoviruses and its product is supposed to act as a pathogenicity factor by suppressing RNA silencing-based antiviral host defenses [21]. We could annotate here the ORF AC5 (with a size of 252 nucleotides) in a wide range of isolates obtained from *P. vulgaris*, *P. lunatus*, *M. lathyroides*, *G. max*, and *M. erythroloma*. Interestingly, two isolates reported infecting *M. lathyroides* (JN419004 and JN419003) displayed the ORF AC5 with a quite different size (=276 nucleotides).

The BLASTn analysis comparing 412 bp sequences from the Rep gene across BGMV-related isolates showed identity levels ranging from 95 to 100% (with a maximum of 20 mismatches) in comparison with the entire set of *bona fide* BGMV strains. This 412 bp sequence was used in the RNAi intron-hairpin construct that resulted in the genetically modified BGMV-resistant bean cultivar from Embrapa [18].

## 4. Discussion

Herein, we assessed the genomic diversity of 146 isolates classified/named as BGMV by comparing their complete DNA–A and DNA–B sequences with the reference viral isolate. The BGMV isolates clustered into four groups, with two of them being discriminated mainly by their original Fabaceae hosts. In addition, SDT analyses indicated that isolates collectively described as BGMV comprise, in fact, two clear-cut groups of isolates. The first group encompasses *bona fide* BGMV isolates (the majority obtained from *P. vulgaris* and from other distinct hosts within the Fabaceae family). The second group is composed of a closely related begomovirus (with DNA–A identities ranging predominantly from 89 to 91% to representative BGMV isolates). This second group of isolates is mainly associated with *P. lunatus* and *M. lathyroides*. The begomovirus–host plant association was not perfect for all isolates. For instance, two isolates from *P. lunatus* were found to be more likely strains of BGMV, displaying nucleotide identities of 94–95% to the reference isolate. However, it is important to highlight that both host classification and sequence annotations available at in the GenBank database might not be entirely accurate across all BGMV-related accessions as previously observed for distinct tomato-infecting Neotropical begomoviruses [22,23].

Moreover, differences for a set of genomic features were also annotated between these two groups of BGMV-related isolates. Due to the molecular and structural features described herein, we concluded that our Group #3 of isolates (formerly described as BGMV) corresponds, more likely, to a novel *Begomovirus* species. The tentative name for this pathogen is lima bean golden mosaic virus (LBGMV), as previously suggested [8]. The reason why these genetically divergent BGMV-related isolates were not previously identified as novel species can be explained by the fact that most of these isolates were characterized before the novel species demarcation rules were established for the genus *Begomovirus* [3]. Thus, with the current taxonomic criteria, these viral variants can now be unambiguously recognized as strains of a new *Begomovirus* species. In this context, the reclassification of a subset begomoviruses available at GenBank with DNA–A genome with less than 91% identity to representative BGMV-related viral lineages seems to be necessary in order to avoid additional misunderstandings and, especially, for improving the classification system of these closely related legume-infecting *Begomovirus* species.

It is interesting to highlight that variability in the expression of symptoms caused by BGMV isolates in *Phaseolus* species has been detected as early as in the 1960s in Brazil [24]. Three distinct whitefly-transmitted diseases were previously identified as “golden mosaic”, “mottled dwarf”, and “crumpling”. These symptoms were so distinctive that Dr. Álvaro Santos Costa (1965) speculated that a putative complex of closely related begomoviruses was inducing these diseases [24]. Therefore, a plausible hypothesis is that one of these distinct diseases could be induced by LBGMV isolates (yet undetected at that time). Thus, it will be interesting to carry out comparative assays inoculating *P. lunatus* and *P. vulgaris* cultivars with BGMV and the putative LBGMV isolates in order to identify if there is a peculiar set of symptoms associated with each virus. So far, isolates of BGMV have been reported on *P. lunatus*, but, to our knowledge, no natural infection of *P. vulgaris* by isolates related to the putative LBGMV has yet been reported.

As previously mentioned, the original description of the putative LBGMV species was established in Brazil in the late 1990s [8] employing only a partial sequence of the DNA–A component (1185 nucleotides), encompassing the *Rep* and CP-coding genes of a single isolate (U92531). Our BLASTn analyses employing this original GenBank accession (U92531) indicated identity levels ranging from 94.36% to 97.91% with a collection of 56 GenBank accessions composed mainly of isolates obtained from *P. lunatus* [10,13], *Phaseolus* sp. [13] and from *M. lathyroides* [9]. The highest identity levels of U92531 to *bona fide* BGMV isolates was 89.74% (e.g., KJ939776), indicating that the isolate described by Faria and Maxwell in 1999 [8] was, more likely, the first report of a novel Fabaceae-infecting *Begomovirus* species distinct from BGMV.

The epidemiological consequences as well as the impact of this viral diversity in classical and biotech breeding programs for begomovirus resistance across distinct *Phaseolus* hosts should be reexamined. The 412 bp sequence from the *Rep* gene, which was used for the RNAi intron-hairpin construct of the genetically modified BGMV-resistant bean cultivar [18], displayed identity levels ranging from 95 to 100% (with a maximum of 20 mismatches) to the entire set of *bona fide* BGMV isolates. These results lead us to the prediction that this transgenic bean cultivar should have a stable and large-spectrum resistant reaction to all isolates sequenced to date.

## 5. Conclusions

We recognized a host-guided pattern of genetic organization of the BGMV-related isolates. In addition, a distinct set of biological as well as virus-specific genomic features of the iterons and Rep-associated protein motifs were detected especially across *P. vulgaris* and *P. lunatus* isolates. All these peculiar host interactions and genomic features indicated that a subset of *P. lunatus*-infecting isolates is not appropriately classified as BGMV strains, but they are more likely isolates of a putative novel *Begomovirus* species. For this putative new species, we suggest adopting the previously proposed name—lima bean golden mosaic virus—LBGMV [8] associated with the tentative binomial name of *Begomovirus fariai*. Hence, for isolates traditionally classified as BGMV, some may in fact represent a distinct species-most likely LBGMV. Our observation underscores the importance of continuous molecular characterization, as the presence of such divergent isolates contributes significantly to our understanding of the genetic diversity within the Fabaceae-infecting *Begomovirus* species. The impact of this host-related viral diversity should now be taken into consideration by classical and biotech-breeding programs for viral resistance in both hosts (*P. vulgaris* and *P. lunatus*).

## Data Availability

The datasets generated during and/or analyzed during the current study are not publicly available as they are part of ongoing research and further analyses but are available from the corresponding author on reasonable request.

## Supplementary Materials

The following supporting information can be downloaded at https://www.mdpi.com/article/10.3390/pathogens14070697/s1; Figure S1: Phylogenetic tree of a set of complete genomes of DNA–A components showing the phylogenetic identities/distances of 146 bean golden mosaic virus (BGMV) isolates available at the GenBank; Figure S2: Pairwise identity analysis with the Sequence Demarcation Tool (SDT) was carried out using the sequence information of the DNA–A component of isolates obtained from *Phaseolus lunatus,* unclassified *Phaseolus* species, and *Macroptilium lathyroides*, in relation to the sequence of the reference (NC_004042) bean golden mosaic virus (BGMV) isolate; Figure S3: Pairwise identity analysis with the Sequence Demarcation Tool (SDT) was carried out using the sequence information of the DNA–A component of isolates obtained from *Glycine max* (Gm), *Macroptilium lathyroides* (Ml), and *M. erythroloma* (Me), indicating their identities in relation to the sequence of the reference (NC_004042) bean golden mosaic virus (BGMV) isolate; Figure S4: Pairwise identity analysis with the Sequence Demarcation Tool (SDT) was carried out using the sequence information of the DNA–B; Figure S5: Common region, iterons, and motifs of the Replication-associated protein (Rep) with the DNA–A and DNA–B sequences of bean golden mosaic virus—BGMV reference (NC_004042) isolate (highlighted in red font color) compared with other isolates with identity levels greater than or equal to 96%; Figure S6: Common region, iterons, and motifs of the Replication-associated protein (Rep) with the DNA–A and DNA–B sequences of bean golden mosaic virus—BGMV reference (NC_004042 and NC_004043), isolate (in red), compared with other isolates with identity levels ranging from 89 to 91%; Figure S7: Symmetric region ACTT–(N7)–AAGT of isolates described as bean golden mosaic virus (BGMV); Table S1: Accession codes and information of bean golden mosaic virus (BGMV) DNA–A sequences used in the work; Table S2: Accession codes and information of bean golden mosaic virus (BGMV) DNA–B sequences used in the work.
