# Supplementary material for: Reexamining a Host-Associated Genomic Diversity of Bean Golden Mosaic Virus (BGMV) Isolates from Phaseolus Species and Other Fabaceae Hosts
Source: Pathogens. 2025 Jul 15;14(7):697. doi: 10.3390/pathogens14070697 (PMC12299109; doi:10.3390/pathogens14070697)
Supplement: Supplementary file 1 [file pathogens-14-00697-s001.zip › Table S1 020725.pdf]

Table S1 Accession codes and information of *Bean golden mosaic virus* (BGMV) DNA-A sequences used in the work

Access number - GenBank DNA-A	Hosts	Reference*
FJ665283	*Glycine max*	Fernandes et al. (2009)
MN822294	*Macroptilium erythroloma*	Batista et al. (2020)
JN419003, JN419004 and JN419006	*Macroptilium lathyroides*	Silva et al. (2011)
KJ939766, KJ939767, KJ939768, KJ939769, KJ939770, KJ939771, KJ939772, KJ939773, KJ939774, KJ939775, KJ939776, KJ939777 and KJ939778	*Macroptilium lathyroides*	Sobrinho et al. (2014)
NC004042 (Reference isolate)	*Phaseolus vulgaris*	Gilbertson et al. (1991)
MG334552	*Phaseolus vulgaris*	Bertholdo; Faria; Coelho (2017) (Direct submission in Genbank)
KJ939792, KJ939793, KJ939796 KJ939798, KJ939799, KJ939800 KJ939802, KJ939804, KJ939809 KJ939810, KJ939815, KJ939819 KJ939820, KJ939791, KJ939794 KJ939795, KJ939797, KJ939801	*Phaseolus vulgaris*	Sobrinho et al. (2014)

KJ939803, KJ939805, KJ939806<br>KJ939807, KJ939808, KJ939811<br>KJ939812, KJ939813, KJ939814<br>KJ939816, KJ939817, KJ939818<br>KJ939779, KJ939780, KJ939781<br>KJ939782, KJ939783, KJ939784<br>KJ939785, KJ939786, KJ939787<br>KJ939788, KJ939789, KJ939790<br>KJ939821, KJ939822, KJ939823<br>KJ939824, KJ939825, KJ939826<br>KJ939827, KJ939828, KJ939829<br>KJ939830, KJ939831, KJ939832<br>KJ939833, KJ939834, KJ939835<br>KJ939837, KJ939838, KJ939839<br>KJ939844, KJ939845, KJ939846<br>KJ939847, KJ939848, KJ939840<br>KJ939841, KJ939842. KJ939843<br>KJ939849, KJ939850, KJ939851<br>KJ939852, KJ939853 and KJ939836		
KJ939721, KJ939709, KJ939725,<br>KJ939726, KJ939727, KJ939728<br>KJ939729, KJ939730, KJ939731<br>KJ939732, KJ939733, KJ939734<br>KJ939735, KJ939736, KJ939707<br>KJ939719, KJ939720, KJ939714<br>KJ939723, KJ939712, KJ939713<br>KJ939708, KJ939716, KJ939717<br>KJ939715, KJ939754, KJ939755<br>KJ939756, KJ939757, KJ939758<br>KJ939759, KJ939760, KJ939761<br>KJ939762, KJ939763, KJ939764	*Phaseolus lunatus*	Sobrinho et al. (2014)

KJ939739, KJ939740, KJ939741 KJ939742, KJ939743, KJ939744 KJ939745, KJ939746, KJ939747 KJ939748, KJ939749, KJ939750 KJ939751, KJ939752, KJ939722 and KJ939753		

*: Complete citation is listed in Reference
