# Supplementary material for: Reexamining a Host-Associated Genomic Diversity of Bean Golden Mosaic Virus (BGMV) Isolates from Phaseolus Species and Other Fabaceae Hosts
Source: Pathogens. 2025 Jul 15;14(7):697. doi: 10.3390/pathogens14070697 (PMC12299109; doi:10.3390/pathogens14070697)
Supplement: Supplementary file 1 [file pathogens-14-00697-s001.zip › Table S2 020725.pdf]

Table S2 Accession codes and information of *Bean golden mosaic virus* (BGMV) DNA-B sequences used in the work

Access number GenBank DNA-B	Hosts	Reference*
NC004043 (Reference isolate)	*Phaseolus vulgaris*	Gilbertson et al. (1991)
MG334553	*Phaseolus vulgaris*	Bertholdo; Faria; Coelho (2017)<br>(Direct submission in Genbank)
JN419017 and JN419008	*Macroptilium lathyroides*	Silva et al. (2011)
MH925107	*Phaseolus lunatus*	Ferro et al. (2018)

*: Complete citation is listed in Reference
