# Supplementary figures and images for: Reexamining a Host-Associated Genomic Diversity of Bean Golden Mosaic Virus (BGMV) Isolates from Phaseolus Species and Other Fabaceae Hosts
Source: Pathogens. 2025 Jul 15;14(7):697. doi: 10.3390/pathogens14070697 (PMC12299109; doi:10.3390/pathogens14070697)

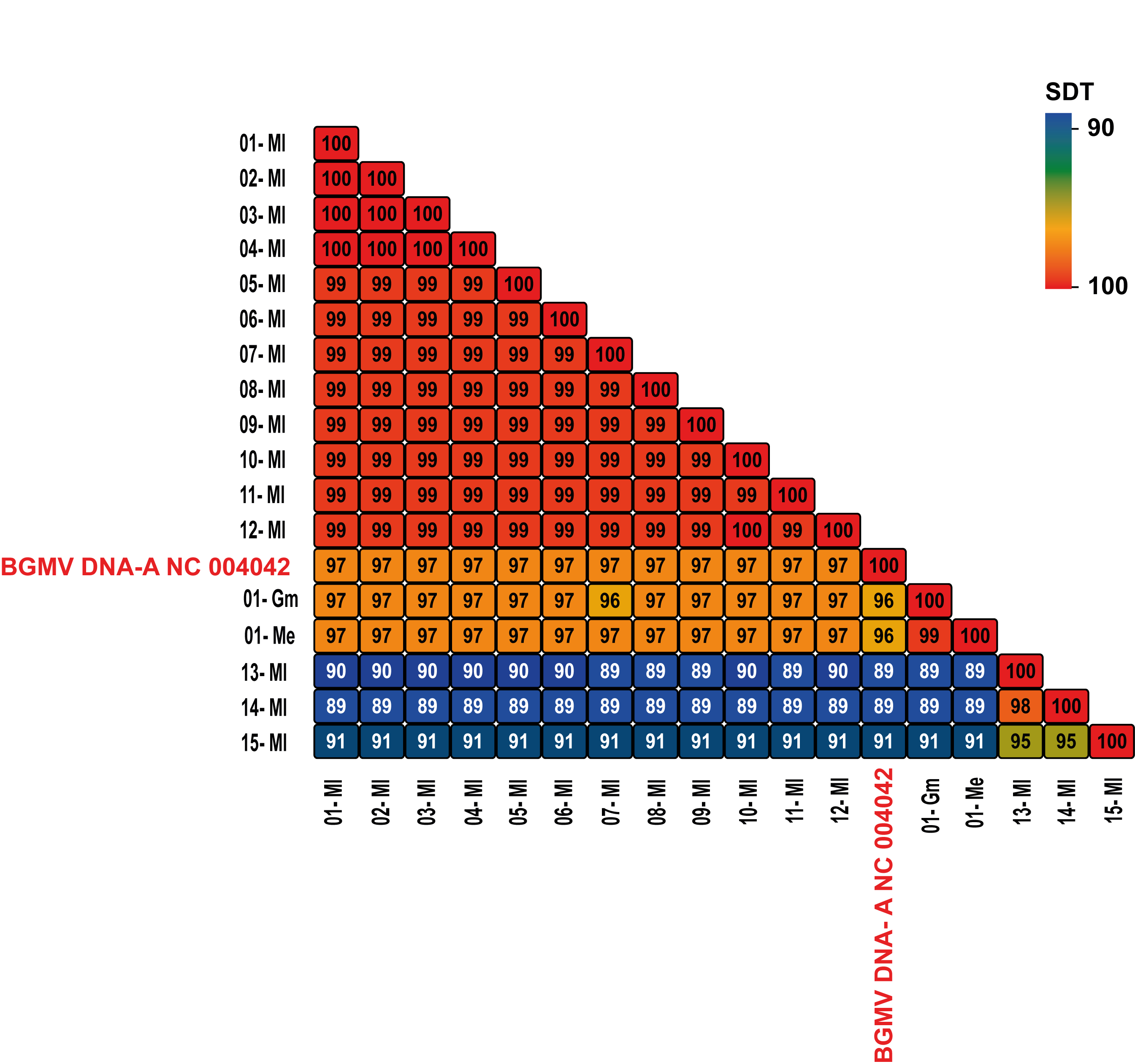

Supplement: Supplementary file 1 [file pathogens-14-00697-s001.zip › Figure S3 070325.tif]

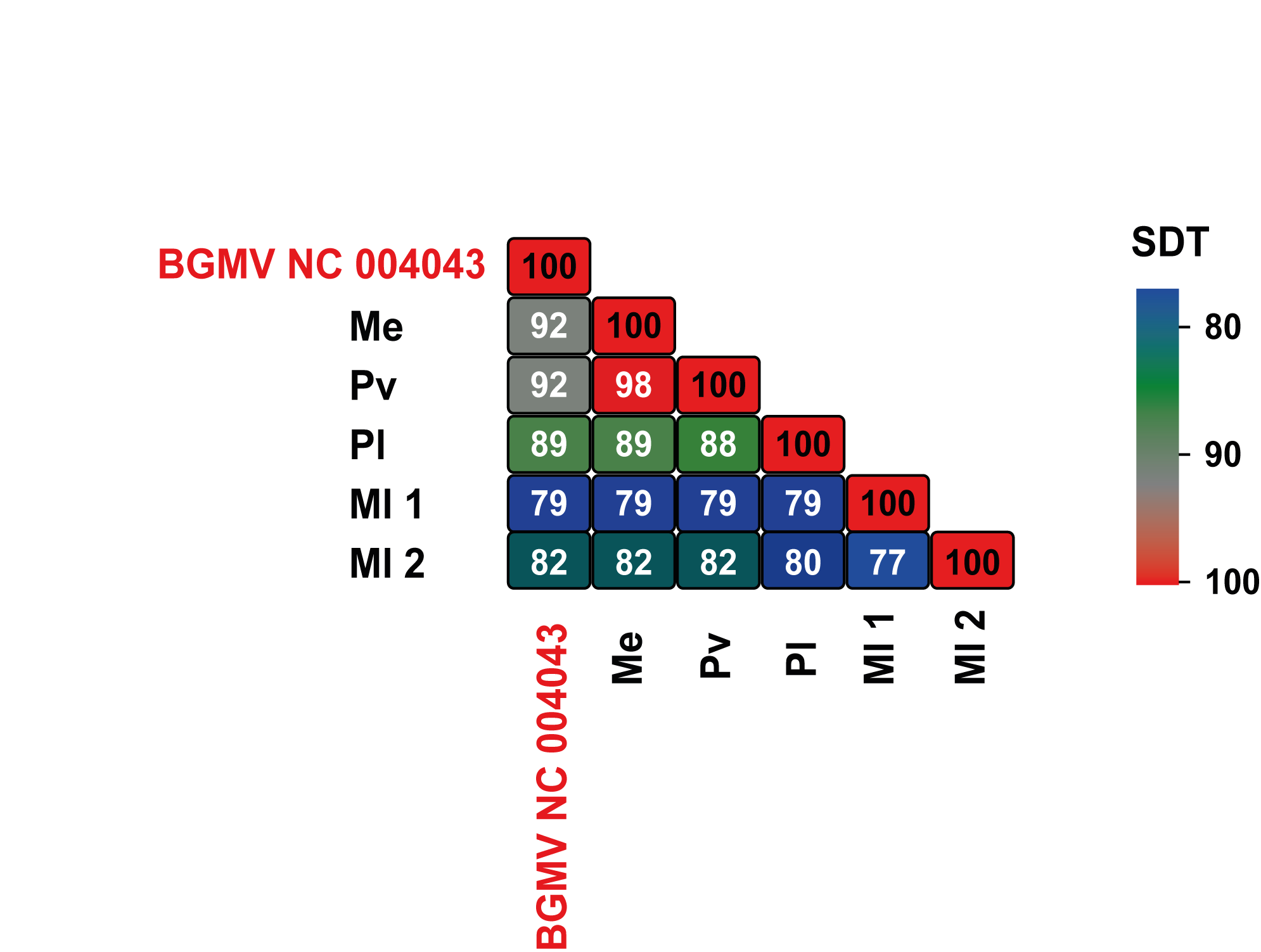

Supplement: Supplementary file 1 [file pathogens-14-00697-s001.zip › Figure S4 070325.tif]

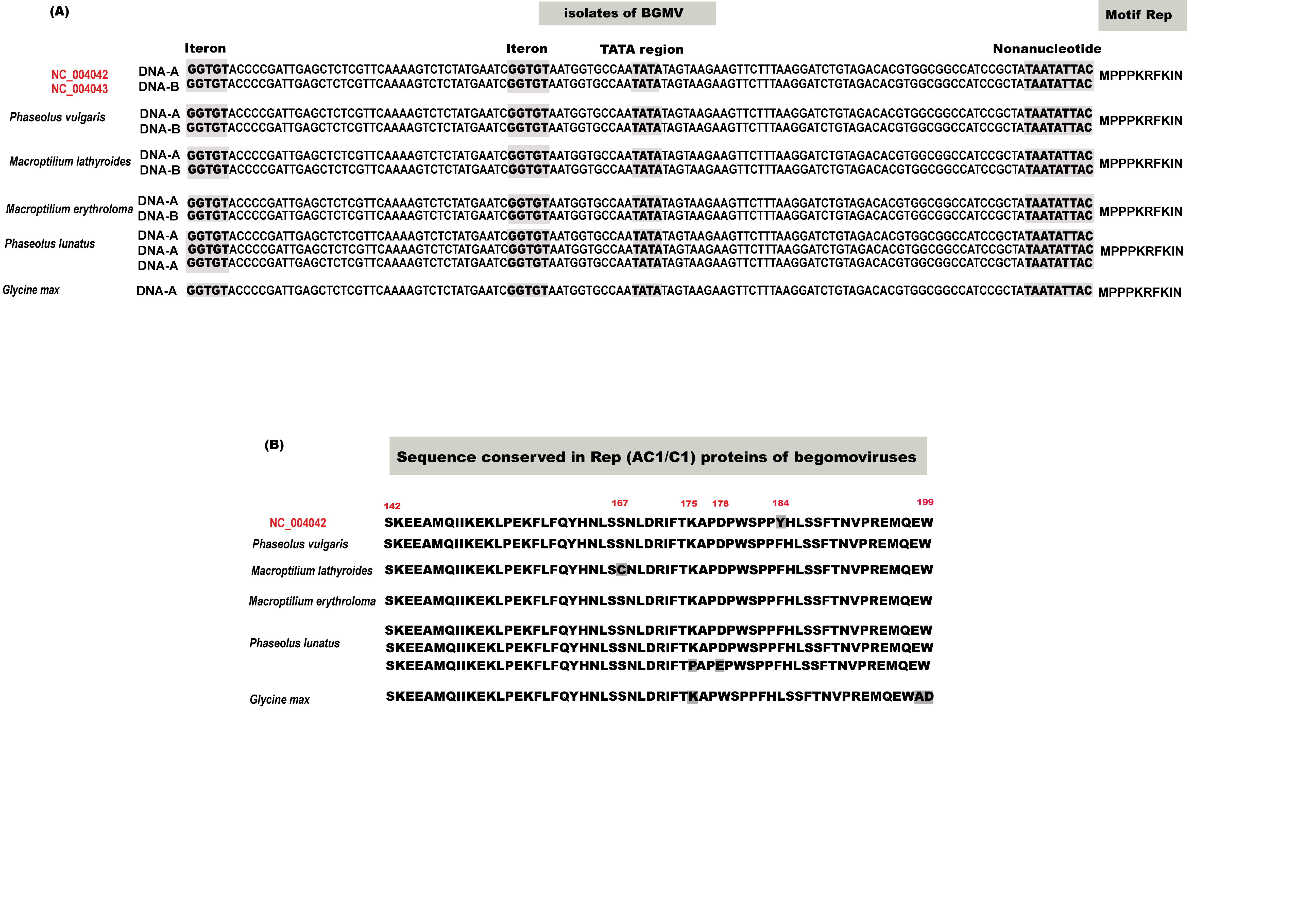

Supplement: Supplementary file 1 [file pathogens-14-00697-s001.zip › Figure S5 070325.tif]

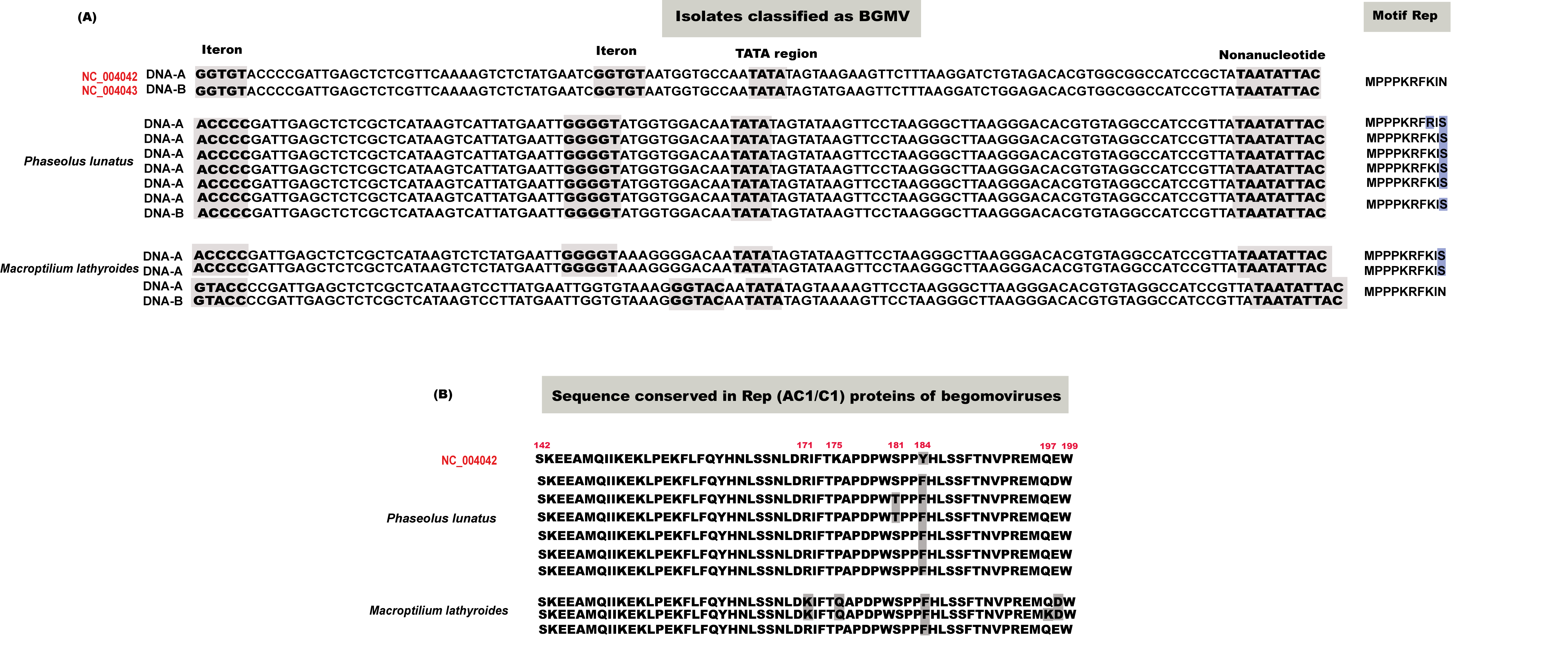

Supplement: Supplementary file 1 [file pathogens-14-00697-s001.zip › Figure S6 070325.tif]

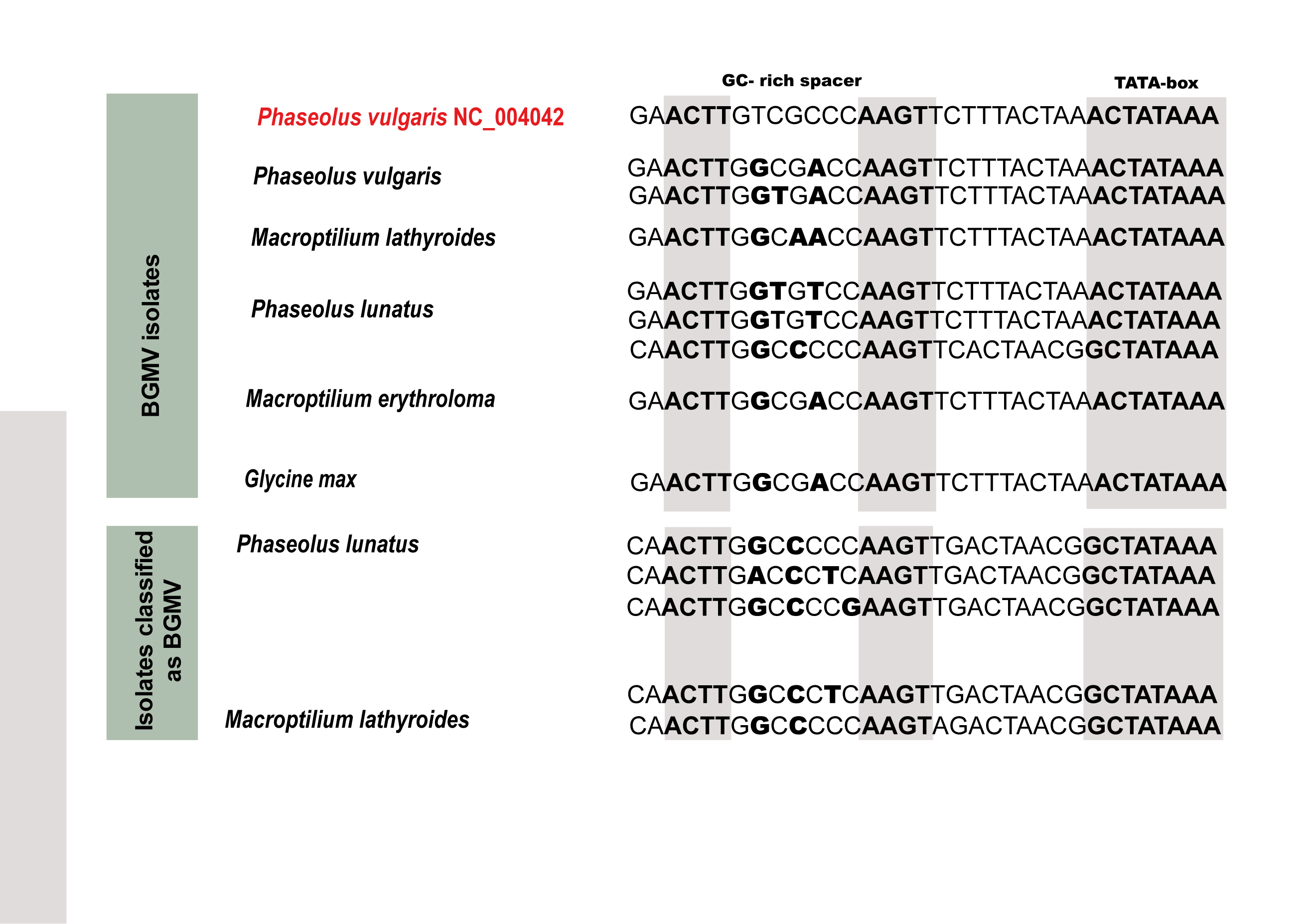

Supplement: Supplementary file 1 [file pathogens-14-00697-s001.zip › Figure S7 070325.tif]

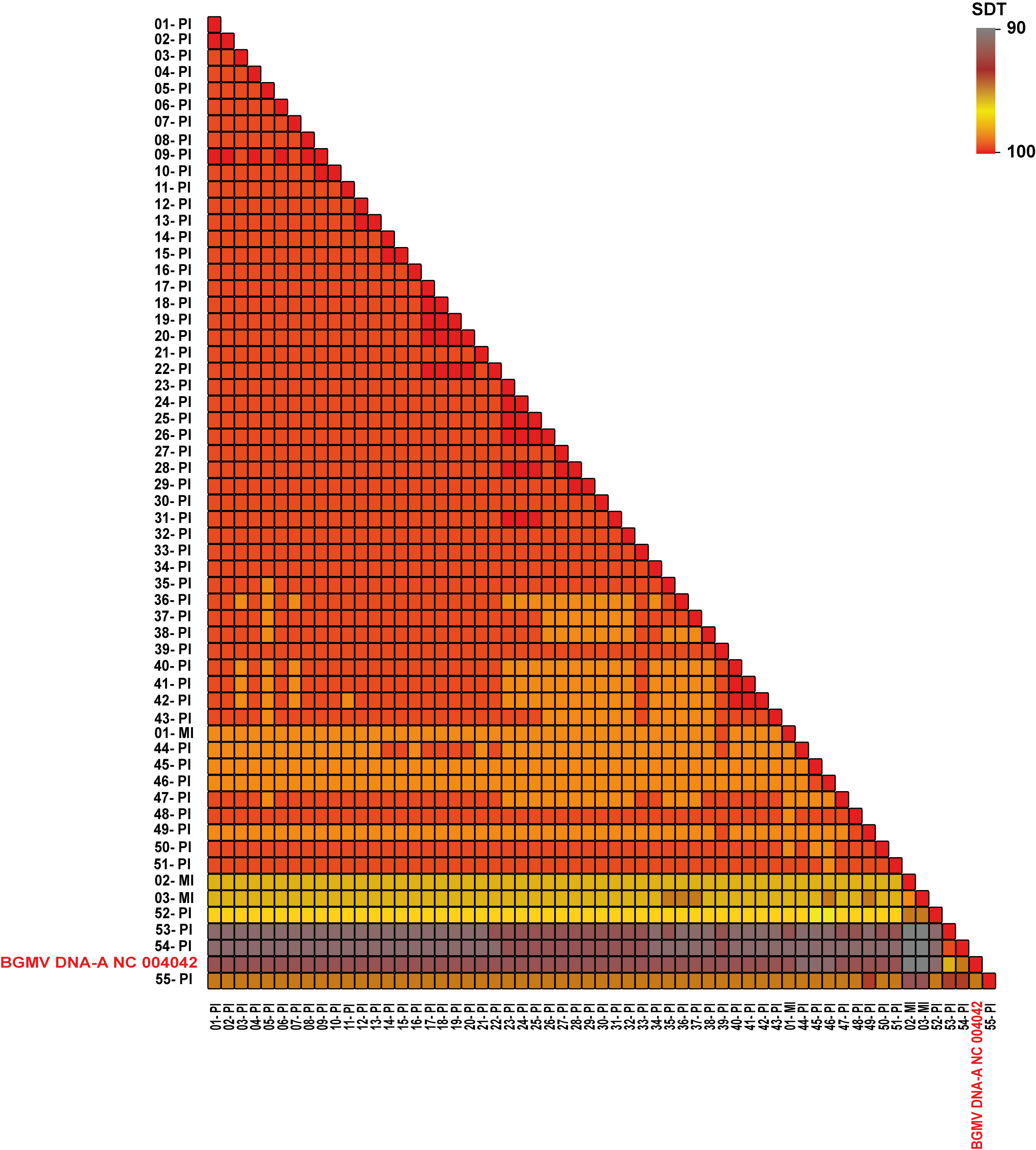

Supplement: Supplementary file 1 [file pathogens-14-00697-s001.zip › New Figure S2 070325.tif]
